# From green to red: Urban heat stress drives leaf color evolution

**DOI:** 10.1126/sciadv.abq3542

**Published:** 2023-10-20

**Authors:** Yuya Fukano, Wataru Yamori, Hayata Misu, Mitsuhiko P. Sato, Kenta Shirasawa, Yuuya Tachiki, Kei Uchida

**Affiliations:** ^1^ Graduate School of Horticulture Sciences, Chiba University, Chiba, Japan.; ^2^ Graduate School of Agricultural and Life Sciences, The University of Tokyo, Tokyo, Japan.; ^3^ Department of Frontier Research and Development, Kazusa DNA Research Institute, Chiba, Japan.; ^4^ Department of Biological Sciences, Tokyo Metropolitan University, Tokyo, Japan.

## Abstract

Prevalence of impervious surface and resulting higher temperatures in urban areas, known as urban heat islands, comprises prominent characteristics in global cities. However, it is not known whether and how urban plants adapt to such heat stress. This study focused on *Oxalis corniculata*, which has intraspecific polymorphism in leaf color (green and red) and examined whether the leaf color variation is associated with urban heat stress. Field observations revealed that green-leaved plants were dominant in green habitats, and red-leaved individuals were dominant in urban habitats, at local (<500 meters), landscape (<50 kilometers), and global scales. Growth and photosynthesis experiments demonstrated that red-leaved individuals performed better under heat stress, while green-leaved individuals performed better under nonstressful conditions. Genome-wide SNP analysis suggests that the red leaf may have evolved multiple times from the ancestral green leaf. Overall, the results suggest that the red leaves of *O. corniculata* observed in cities worldwide are evidence of plant adaptive evolution due to urban heat islands.

## Teaser

A study from Tokyo reports the first evidence of ongoing evolution of urban plants against urban heat-island.

## INTRODUCTION

Urban environments are human-created habitats that occupy approximately 3% of the world’s total land area and often impose several biotic and abiotic selective pressures on urban organisms (*1*–*3*). These urban ecosystems provide valuable opportunities for understanding the processes and impacts of extreme environmental changes on the rapid evolution of plants (*2*, *4*–*7*). Understanding the evolutionary processes that occur in response to environmental stresses in urban areas may be important for understanding the eco-evolutionary dynamics occurring between nature and human societies (*8*–*12*). It may also offer unique opportunity to predict the adaptation of wild and domesticated species in response to extreme environmental conditions, particularly in the face of ongoing and projected global climate change (*13*, *14*).

One of the most pervasive environmental changes in urban habitats is the prevalence of impervious surfaces, such as asphalt, concrete, brick, and stone, which cover much of habitats’ ground surface (*15*–*17*). These surfaces are highly efficient at absorbing and generating heat, leading to higher temperatures in urban areas, known as urban heat islands (*16*, *18*). Such high temperatures can have severe ecological impacts on the behavior, physiology, and life history of various organisms, including humans that live in cities (*19*–*23*). While the evolutionary effects of urban heat stress on some animals have begun to be explored (*24*–*29*), the evolutionary impacts on plants have not yet been examined. Plants, which are sessile organisms highly sensitive to environmental stresses related to the ground surface (*20*, *21*, *30*, *31*), are likely to be under strong selection pressure from urban heat islands. Given the diverse evolutionary responses observed in urban plants (*7*, *12*, *32*, *33*), it is possible that they are also rapidly adapting to heat islands. In this work, leaf color variation of an *Oxalis* plant was studied to explore the evolutionary impacts of urban heat islands on local plants.

**Fig. 1.**
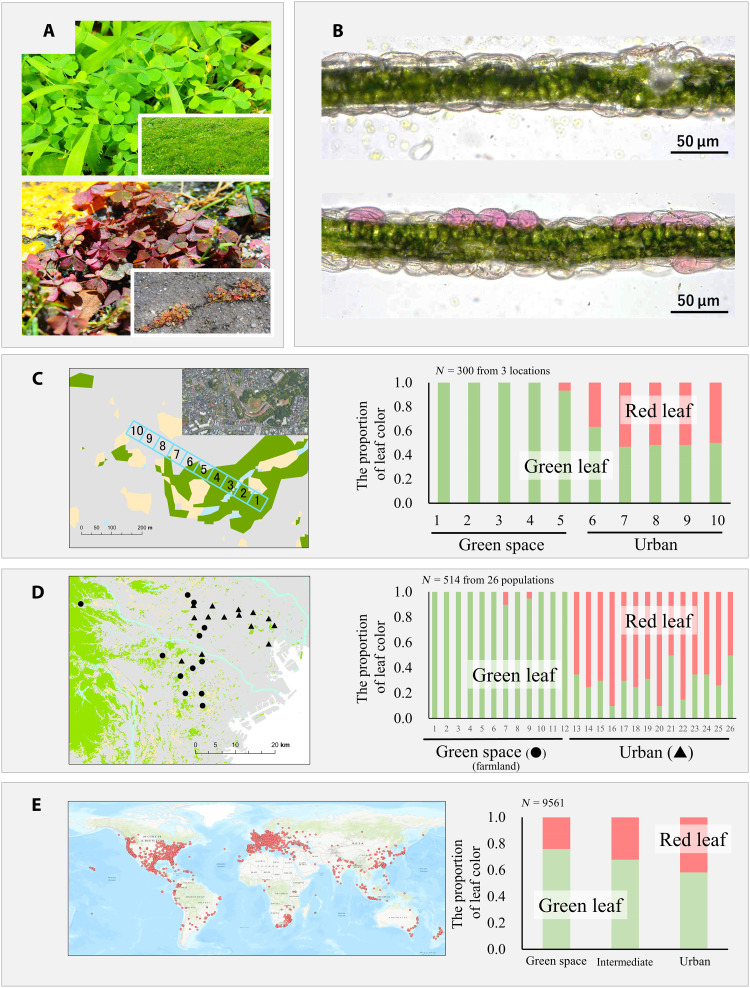
The morphology and distribution of green- and red-leaved *O. corniculata*. The representative pictures of green- and red-leaved individuals in field environments (**A**), microscopic image of a section of green and red leaf (**B**), leaf color distribution of *O. corniculata* between urban and green space (farmland or green sites) at different research scale local levels (**C**), landscape level (**D**), and global level (**E**). The images on the left show the observation sites at each of the survey scales [for (C), one of the three survey locations]. Images on the right show the proportion of individuals for each leaf color. The top right photo in local level picture (C) has been modified from Google Maps. The five-point scale for leaf color has been dropped by two units to improve visibility (1 and 2 as green and 3, 4, and 5 as red).

The creeping woodsorrel, *Oxalis corniculata* L., is a cosmopolitan plant that has the third largest distribution of vascular plant species worldwide (*34*). The species grows in various types of habitats throughout the urban-rural gradient, such as riverbanks, lawns, parks, roadsides, railway tracks, seminatural grassland, and farmland (*35*). It exhibits interspecific variation in leaf color, with some individuals having more common green leaves (*O. corniculata* L.) and individuals with reddish or purplish leaves (*O. corniculata* L. *f. atropurpurea* and *O. corniculata* L. *f. rubrifolia*, referred to hereafter as green and red leaves, respectively; Fig. 1A) (*36*, *37*). Unlike most red leaves commonly seen during abscission in deciduous trees and in young developing leaves during spring and summer, the red leaves of this species are not typically induced by environmental heat stress but continually exhibit such a constitutive trait (fig. S1). Thus, the green-red leaf variation is a genetically determined natural intraspecific variation. *O. corniculata* shows creeping growth habit, and in cities, it grows on impermeable surfaces such as road gaps (Fig. 1A) and is therefore subjected to severe heat stress, particularly in summer.

We noticed that red-leaved individuals were observed to be commonly found on impervious surfaces in urban areas but rarely found in farmland areas or green spaces within cities (Fig. 1A). Several studies on inducible leaf reddening (e.g., winter leaves) suggest that such a color change has an adaptive role in light attenuation and antioxidant protection during environmental stresses (*38*–*43*). Receiving bright light under environmental stress, such as cold, heat, and dry conditions, can result in excess energy capture relative to photosynthetic ability. The excess sunlight energy causes photoinhibition of photosynthesis, formation of reactive oxygen species (ROS), and greater photooxidative damage (*44*). Red pigments in leaves (e.g., anthocyanin) are thought to alleviate these stress conditions by two different physiological mechanisms: intercepting light (light attenuation) and/or neutralizing ROS directly via antioxidants (*38*–*43*). Urban ground surfaces, which are mostly characterized by being impermeable, are subject to higher temperatures than green surfaces (fig. S2). On the basis of the adaptive role of red leaves, urban heat islands may cause plant stress in *O. corniculata*, leading to photoinhibition, which red leaves can help alleviate. The high frequency of red-leaved individuals in urban areas may be due to rapid adaptive evolution in response to urban heat islands.

To explore this evolutionary hypothesis, three different approaches were combined: (i) comparing leaf color distribution between urban and nonurban settings at local, landscape, and global scales; (ii) quantifying the adaptive benefits and costs of green and red leaves by comparing biomass growth and photosynthetic ability under heat-stress and nonheat conditions between green and red leaves; and (iii) estimating evolutionary scenarios of red leaves through genome-wide single-nucleotide polymorphism (SNP) analysis. Overall, our results suggest that the red leaf of *O. corniculata* in urban areas is evidence of rapid adaptive evolution in response to urban heat islands, making it one of the most discernable and familiar examples of adaptive evolution in cities worldwide.

## RESULTS

The detailed results of all statistical analyses are summarized in table S1.

### Morphology and pigment

Optical microscopy showed that the epidermal tissue of red-leaved *O. corniculata* was reddish (Fig. 1B). Chlorophyll and anthocyanin contents were higher in red-leaved than in green-leaved plants, regardless of growing conditions (fig. S1). The spectrum of the red pigment extract showed an absorption peak at 528 nm, which was in consistent with that of anthocyanin (530 to 560 nm) (*45*).

### Field observations

Local (<500 m), landscape (<50 km), and global-scale field observations were undertaken to quantify the differences in green-red leaf variation between urban and green settings (table S2). For the local-level observation, the transect surveys across urban and green space in the three locations near Tokyo showed a clear difference in the distribution of green and red leaves between urban and green space (Fig. 1C). Green-leaved individuals were dominant in green spaces, and the frequency of red-leaved individuals was higher in urban spaces. For the landscape-level observation, the distribution of green- and red-leaved individuals showed the same trends (Fig. 1D); green-leaved individuals were dominant in farmland populations, and the frequency of red-leaved individuals was higher in urban populations. Global field observations collected from iNaturalist citizen science data (https://inaturalist.org) also showed the same trends (Fig. 1E). The images of *O. corniculata*, taken at sites with more impervious surfaces (urban sites), were more likely to be of red-leaved plants, while those taken at sites with less impervious surfaces (green sites) were more likely to be green-leaved.

### Growth under nonstress and stress conditions

To determine whether the effects of heat stress treatments on biomass growth differed by leaf color under both controlled and uncontrolled conditions, we examined the effects of the interaction between the type of leaf color and stress treatments. Since the interaction term was statistically significant in both conditions (table S1), we subsequently investigated the effect of leaf color on biomass growth under stress and nonstress treatments separately.

**Fig. 2.**
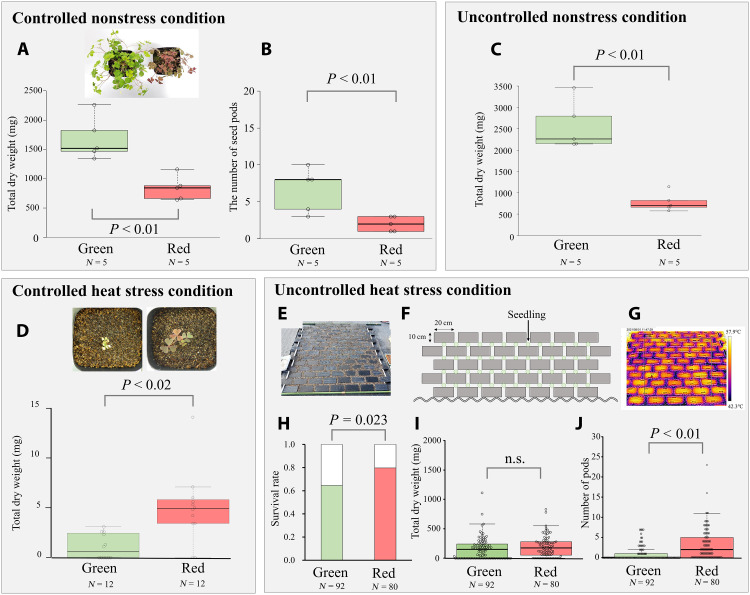
The results of growth experiments under nonstress and stress conditions. The comparisons of total dry weight and seed pods between green- and red-leaved individuals of *O. corniculata* after 7 weeks of cultivation under controlled condition [grow chamber (**A** and **B**)], 3 months of cultivation under uncontrolled nonstress condition [greenhouse (**C**)], and 4 weeks of cultivation under uncontrolled heat stress condition [growth chamber (**D**)]. Experimental setting of uncontrolled heat stress condition [brick pavement (**E** and **F**)] with example of surface temperatures of the plot measured on sunny days. (**G**) The comparisons of survival rate, (**H**) total dry weight, (**I**) and the number of pods. (**J**) Between green- and red-leaved individuals after 6 weeks of cultivation under uncontrolled heat stress conditions. Box plots represent median (horizontal line), 25th and 75th percentiles (box), and 10th and 90th percentiles (whiskers), and each dot represents an individual value. n.s., not significant.

Under nonstress growth conditions, green-leaved plants had a higher growth rate than red-leaved ones. Under the controlled condition treatment (growth chamber, 25°C with 60% humidity), the total dry weight of green-leaved plants was larger than red-leaved plant dry weight after 4- and 7-week growth (fig. S3 and Fig. 2A, respectively). After 7 weeks of cultivation, the number of seed pods of green-leaved individuals was higher than that of red-leaved individuals (Fig. 2B). In the uncontrolled condition treatment (greenhouse, 15° to 30°C), the total dry weight of green-leaved plants was larger than those of red-leaved plants after 3 months of cultivation (Fig. 2C).

In contrast, under heat-stress conditions, red-leaved plants had a higher growth rate than green-leaved ones. In the controlled heat-stress condition (growth chamber, 35°C with 60% humidity), red-leaved plants had a larger total dry weight than green-leaved ones after 4 weeks of cultivation (Fig. 2D and fig. S4). Seedlings grown in the brickyard plot simulating an urban roadside endured extremely high surface temperatures (Fig. 2, E to G), and red-leaved plants had lower mortality (Fig. 2H) and produced more seed pods (Fig. 2J) than green-leaved ones. There was no significant difference in total dry weight (Fig. 2I) between green- and red-leaved plants.

### Photosynthesis under nonstress and stress conditions

**Fig. 3.**
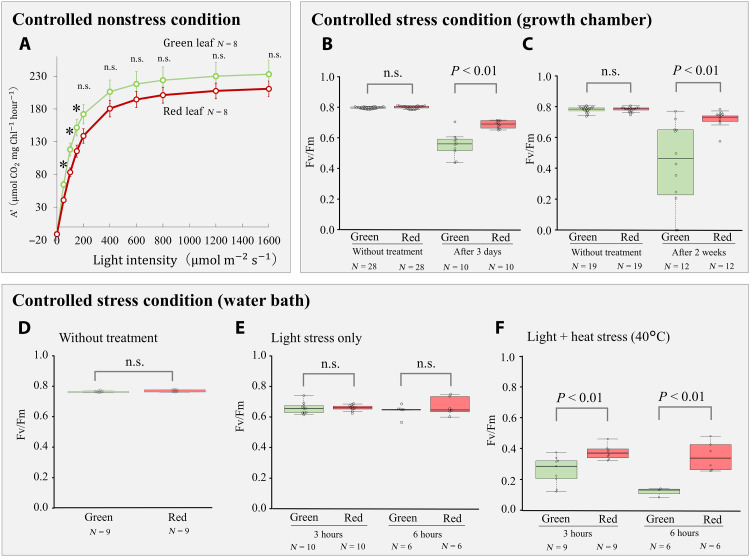
The results of photosynthetic experiments under nonstress and stress conditions. The comparisons of the light response curve of photosynthesis per chlorophyll between green and red leaves of *O. corniculata* [(**A**), means ± SE, asterisks indicated the significant differences, *P* < 0.05]. The comparisons of maximum photochemical efficiency of photosystem II (Fv/Fm) between green and red leaves without and after 3 days (**B**) and without and after 2 weeks (**C**) under controlled heat stress conditions. The comparisons of Fv/Fm between green and red leaves in the water bath experiment without and after 3 and 6 hours of controlled heat and light stress treatment: without treatment (**D**), light stress only (**E**), and light and heat stress (**F**). Box plots represent the median (horizontal line), 25th and 75th percentiles (box), and 10th and 90th percentiles (whiskers); each dot represents an individual value.

Under nonstress conditions, the light response curve of photosynthesis per chlorophyll showed a different pattern for green and red leaves (Fig. 3A). At low light intensity (<150 μmol m^−2^ s^−1^), green leaves had higher photosynthetic rates than red leaves (significant interactions between light level and the type of leaf color), while there was no significant difference at light intensities greater than 200 μmol m^−2^ s^−1^.

In contrast, under heat stress, red leaves had a higher photosynthesis rate than green leaves in both long-term (growth chamber) and short-term (water bath) stress treatments. Without the stress treatment, there were no differences in Fv/Fm (quantum efficiency of photosystem II) between the green and red leaves (fig. S5). After 3 days and 2 weeks of heat stress in a growth chamber, the Fv/Fm of green leaves was greatly reduced compared to that of red leaves (Fig. 3, B and C).

The water bath experiment used to examine the influences of temperature and light stress on the photosynthetic efficiency of green and red leaves found a significance for the leaf color × heat stress interaction but not for the leaf color × light interaction, indicating that only temperature stress had a significant effect on the differences in photosynthesis between different leaf colors. There were no differences in Fv/Fm between green and red leaves both without stress (Fig. 3D) and after light stress only (Fig. 3E). In contrast, Fv/Fm of green leaves was greatly reduced compared to that of red leaves after experiencing both high (40°C) and low temperature stress (5°C) (Fig. 3F and fig. S6).

### Population genetic analysis

**Fig. 4.**
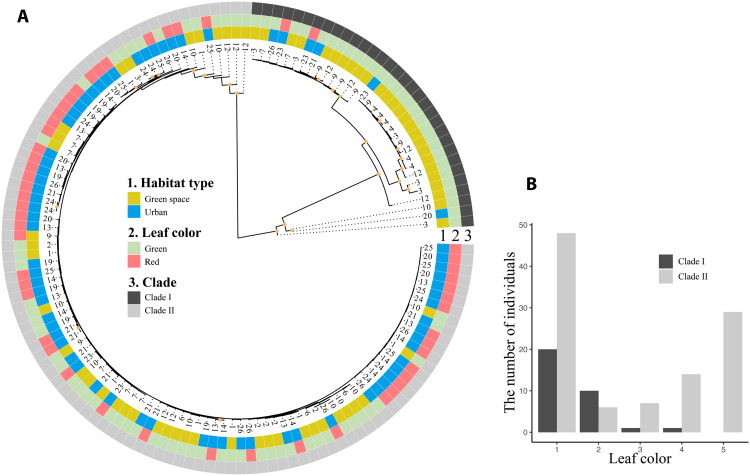
Phylogenetic analysis for landscape-scale observation around Tokyo metropolis. Maximum likelihood tree of 136 individuals with 5282 SNPs (**A**). The number of tip labels indicates population ID, habitat type, leaf color, and clade are shown in external circles. The five-point scale for leaf color is dropped by two units to improve visibility (1 and 2 as green and 3, 4, and 5 as red). Distribution of leaf color level in clades I and II (**B**). The yellow circles on the tree indicate the nodes with bootstrap values greater than 50.

A total of 5282 SNPs were detected across 136 individuals in landscape-scale observations. Phylogenetic analysis revealed two clades in the tested population with high levels of support (Fig. 4A). Clade I included 32 individuals, while clade II included 104 individuals. The average leaf color scores of clades I and II were 1.47 and 2.71, respectively. The distribution of the scores in clade I was monomodal, whereas that in clade II was bimodal (Fig. 4B). Genetic differentiation was not explained by location or habitat type in the individuals through principal components analysis (PCA), isolation by distance (Mantel’s test, *P* > 0.05), or population structure (fig. S7 and table S3).

## DISCUSSION

Although dominance of impermeable surfaces and their resulting urban heat islands are prominent characteristics of cities, the adaptive evolution of plants to these characteristics has not been thoroughly investigated. This study found the strong association between red leaf phenotype in *O. corniculata* and the increase of impermeable surfaces, such as asphalt and concrete roads in urban areas (Fig. 1). Growth and photosynthesis experiments demonstrated that red-leaved individuals have higher growth rates and photosynthetic efficiency under high temperature stress compared to green-leaved ones, while green-leaved plants display higher growth rates and photosynthetic efficiency under nonstressful conditions (Figs. 2 and 3). These results indicate the adaptive benefit and cost of red leaves associated with heat stress and suggested that the red leaf phenotype in *O. corniculata* evolved as an adaptation to heat stress caused by urban impermeable surfaces. Urban impervious surfaces cause plant heat stress, reducing their photosynthetic capacity due to photoinhibition and resulting in decreased biomass growth; the red pigment in the leaves may alleviate this stress. Such surfaces create strong thermal and drought stress, while plant densities are generally lower and plant-plant competition mitigated (*46*). In such habitats, red leaves with high stress tolerance and low competitiveness (i.e., low photosynthetic efficiency and growth rate) are advantageous. In contrast, in green spaces with high plant density and strong competition, green leaves with low stress tolerance and high competitiveness (high photosynthetic efficiency and growth rate under nonstress conditions) will be favored. Extending Grime’s competition–stress–ruderality (CSR) Triangle theory (*47*) to eco-evolutionary dynamics, the urban-rural gradient may cause strong divergent selection, favoring stress-tolerant phenotypes in urban populations and competitive phenotypes in rural populations.

Genome-wide population genetic analysis provided further insight into the evolutionary history of the red leaf phenotype. At the landscape level, *O. corniculata* populations did not exhibit any evidence of population structure (e.g., no evidence for isolation by distance, fig. S7A), and the variation in red and green leaves did not align with population genetic structure (fig. S7B). This suggests a large amount of gene flow among different populations and between red- and green-leaved individuals. *O. corniculata* has small seeds with ballistic dispersal, and these seeds are easily transferred by humans and soil movements. Such characteristics would facilitate high genetic exchange between populations. Despite the high genetic exchange among populations, there was a clear difference in the leaf color phenotype between urban and green settings within tens or hundreds of meters (Fig. 1, C and D), suggesting a strong divergent selection of leaf color between them. The lack of distinct phylogenetic clusters of red-leaf individuals would rule out an evolutionary process in which the red phenotypes observed in urban populations have recently emerged in a particular population and subsequently spread to various other urban populations. In contrast, the proportion of red/green leaves differed between the clades (Fig. 4), with clade II having a higher proportion of reddish leaves than clade I (Fig. 4B). This result suggests that the evolutionary pattern of red leaves from ancestral green leaves differs among clades and that individuals in clade II might share a genomic basis for red leaves to evolve more readily. Further research, including a detailed analysis of the genome and identification of the genetic basis for the red leaf trait, is needed to fully understand its evolutionary history. While it has been proposed that red-leaved varieties are of horticultural origin (*36*), the existence of a green-red variation was reported in an older document from Japan (*48*) where there is no record of horticultural cultivation of this species, suggesting that the green-red variation may be a natural polymorphism. Although red and green leaves are recognized as different forma, our results indicate that both types belong to the same paraphyletic group. However, the mechanisms by which this variation is maintained in pre-urban environments remain unknown. Red-leaved individuals may have had an advantage in natural habitats with high temperatures and low plant density, such as bare land and riverbanks, as has been reported for red-leaved individuals of *Setaria viridis* frequently observed in riverside habitats (*49*).

Anthocyanins may be the key secondary metabolite for adaptation to the urban heat island. Under heat stress conditions, the rate of CO_2_ fixation in the chloroplast’s Calvin cycle is reduced compared to the rate of photosynthetic electron transport, and thus, excess energy and reduced form of nicotinamide adenine dinucleotide phosphate (NADP^+^) can be accumulated (*50*). When plants are exposed to intense light under these conditions, electrons from photosystem II are not transferred to NADP^+^ and instead are transferred to oxygen, resulting in the generation of ROS that can damage chloroplasts and reduce photosynthesis efficiency. Anthocyanins may help mitigate the damage caused by ROS through light attenuation and antioxidant protection (*38*, *39*, *41*, *51*). While the light attenuation effect occurs when epidermal anthocyanins absorb green and ultraviolet-B light, the antioxidant protection occurs when anthocyanins in the leaf flesh tissue directly remove ROS (*51*–*53*). In the red leaves of *O. corniculata*, anthocyanins were concentrated in the epidermis (Fig. 1B), suggesting light attenuation by epidermal anthocyanins rather than antioxidant protection. The red leaves of *O. corniculata* may have an adaptive function in mitigating photoinhibition caused by excessive light under heat stress in urban settings through light attenuation.

The stress-induced accumulation of anthocyanins is a common plastic response throughout the plant kingdom, and constitutive red leaves—those that accumulate pigment even in the absence of stress—are known in many other cultivated and wild species (*40*, *42*, *54*). However, the evolutionary dynamics of the red leaf phenotype are not well understood (*40*, *42*, *54*). The natural variation of leaf color in *O. corniculata* can serve as a model system for studying leaf color evolution, as the species is small in size, is distributed worldwide, and has a rapid life cycle, which allows for experimentation in both laboratory and field conditions. Understanding the eco-evolutionary dynamics between heat stress and leaf color evolution may also have important implications for predicting and mitigating the evolution of wild species and crop breeding strategies in the face of ongoing and future global warming. For example, under high temperature stress, red leaf varieties may show higher population growth rate than green leaf varieties, at the expense of photosynthetic efficiency in weak light.

Although a series of hypotheses suggest that red and yellow leaves have defensive and signal functions against herbivores (*54*, *55*), this function would not apply to the red leaves of *O. corniculata*. In general, the diversity and abundance of herbivores are reduced in urban habitats compared to rural and natural habitats (*56*). Thus, the red leaf evolution of *O. corniculata* should be less affected by selective pressures from herbivores compared to those from environmental stresses. In contrast, leaf color is known to influence the host selection of herbivores (*54*). As a spillover effect of evolution in response to urban heat stress, dominance of red leaves may affect the host utilization and population dynamics of the relatively small number of herbivores in urban settings, such as the pale grass blue butterfly, *Zizeeria maha*.

In conclusion, the results suggest that the red leaf of *O. corniculata* evolved as an adaptation to urban heat islands. In recent years, much attention has been paid to the adaptive evolution of urban organisms as illustrations of eco-evolutionary relationship between humans and urban-dwelling organisms (*2*, *4*–*7*). It is unexpected that the evolutionary response of plants to urban heat islands has not been studied more extensively, given the large number of plants affected by urban heat stress. Adaptive traits to high temperature stress are likely not limited to leaf color, and future studies should focus on a variety of traits to understand plant adaptation to urban heat islands. Understanding the adaptive impact of urban heat stress will also contribute to understanding adaptations required in response to predicted global warming.

## MATERIALS AND METHODS

### Plant morphology and pigment

To examine the distribution of pigment in leaves, leaf sections of green and red leaves were observed under an optical microscope (Fig. 1B). To identify the pigments involving the green-red leaf variation, the amount of chlorophyll and anthocyanins were quantified for plants grown under several nonstress and heat-stress conditions (see fig. S1). Absorbance spectra of anthocyanins were quantified.

### Field observations

Local-, landscape-, and global-scale field observations were undertaken to quantify the spatial distribution of green-red leaf variation in *O. corniculata.* The detail of survey locations are described in table S1. For the local-scale observation, 50 m–by–500 m belt transect surveys were conducted across urban and green spaces in three locations around Tokyo (Fig. 1C, left). The belt transect was divided into 10 plots of 50 m–by–50 m size, with five plots assigned to each urban and green space. In this survey, we began our observations at the border of each plot and recorded leaf color until 10 individuals were observed. In each plot, the colors of the first 10 individuals were recorded on a five-point visual scale (fig. S8), which was highly correlated with the actual anthocyanin contents (fig. S9). In the preliminary complete enumeration survey, we confirmed that the first 10 individuals were sufficient for estimating the overall green/red ratio in each plot. For the figure illustrations, the five-point scale is dropped by two values to improve visibility (scale 1 and 2 as green and 3, 4, and 5 as red). Landscape-scale observations were made at 12 farmland (nonurban populations) and 14 urban sites (urban populations) around the Tokyo metropolis (Fig. 1D, left). We took the approach of increasing the survey area in concentric circles (from a minimum radius of 50 m to a maximum radius of 500 m) from the starting point of the survey (a station for urban areas or a predetermined location on a map for farmland areas). The color of the first 20 individuals of each location were recorded on a five-point visual scale. The leaves of each individual were collected for population genetic analyses. To examine the relationship between urbanization and leaf color variations globally, the citizen science database was used, and images of *O. corniculata* leaves with geographical locations were collected through the iNaturalist platform (https://inaturalist.org). iNaturalist is a one of the largest web- and mobile-based citizen science platforms for documenting biodiversity worldwide. All available observational data for *O. corniculata* submitted before 18 July 2021 (date of data collection) were collected. All images were manually checked and visually rated for leaf color on the five-point scale. The geographical information from the images was used to check the type of shooting location (green or urban space) on Google Maps (Fig. 1E, left). The type of location was determined visually, based on the percentage of impervious surfaces within 20 m^2^ of each location (for urban space, impervious surface defined as >75%, green space <25%, and intermediate between 25 and 75%) (*57*). Images with no leaves or data with anomalous location information (e.g., on sea surfaces) were excluded. In total, 9561 observations were used for analyzing the relationship between leaf color and the type of location.

### Growth experiments under nonstress and stress conditions

Seeds of green- and red-leaved *O. corniculata* were collected from farmland or roadsides within or near the Institute of Sustainable Agro-ecosystem Services (ISAS), the University of Tokyo, Japan (35.43′N, 139.32′E). The colors of green and red leaves determined in the experiment corresponded to one and five, respectively, on the five-level color scale. The seeds were germinated in a 128-well seedling plate under greenhouse conditions, and the seedlings were then transplanted after 2 weeks into small pots (6.5 cm in diameter, 9.8 cm depth). One week after transplanting, seedlings were assigned to each of experimental growth conditions.

### Controlled nonstress condition (growth chamber)

To compare the growth under non–heat stress condition, seedlings were grown for 4 or 7 weeks in growth chambers (LPH-411SPC, Nippon Medical and Chemical Instruments, Osaka, Japan), set at 25°C with 60% humidity and 400 μmol mol^−1^ CO_2_ under a 16-hour light/8-hour dark photoperiod with a light intensity of 150 μmol m^−2^ s^−1^. After 4 and 7 weeks of cultivation, the aboveground, belowground, and total dry weight of plants were measured, and the number of pods of each individual was counted.

### Uncontrolled nonstress condition (greenhouse)

The comparison of the growth under nonstress condition was performed in uncontrolled greenhouse conditions. Seedlings were grown for 3 months in the greenhouse at ISAS. During the growing season from 28 March to 30 June, the temperature range in the greenhouse was 15° to 30°C, relative humidity 30 to 70%, with a maximum light intensity of 1500 μmol m^−2^ s^−1^. After 3 months of cultivation, the aboveground, belowground, and total dry weight of each individual were measured.

### Controlled stress condition (growth chamber)

Seedlings were grown for 4 weeks in the growth chambers with heat stress (35°C with 60% humidity and 400 μmol mol^−1^ CO_2_ concentration under a 16-hour light/8-hour dark photoperiod with a light intensity of 100 μmol m^−2^ s^−1^). After 4 weeks of cultivation, the total dry weight of each individual was measured. Because of the small plant size, we did not measure aboveground and belowground biomass separately.

### Uncontrolled stress condition (brick pavement)

Uncontrolled natural stress conditions were achieved by making an artificial brick pavement plot with 100 brick blocks (10 cm by 20 cm by 4 cm) arranged in 15 brick rows of 7 to 8 bricks each, spaced 4 cm apart. The gaps between the bricks were filled with commercial soil. A total of 180 individuals (96 green-leaved and 84 red-leaved) were transplanted into the plot on 26 July 2021 and cultivated until 6 September (6 weeks). Water was supplied on most days. After 6 weeks, the number of surviving plants and seed pods for each individual was counted. The total dry weight of each plant was measured after washing and drying.

### Photosynthetic experiments under nonstress and stress conditions

#### Controlled nonstress condition (growth chamber)

To compare the photosynthetic capacity of green and red leaves under nonstress conditions, CO_2_ assimilation rate was measured in plants grown at 25°C with 60% humidity and 400 μmol mol^−1^ CO_2_ concentration under a 16-hour light/8-hour dark photoperiod and a light intensity of 150 μmol m^−2^ s^−1^ with an open gas exchange system (LI-6400XT, LI-COR, Lincoln, Nebraska, USA). The light response curve of CO_2_ assimilation rate was measured under the same conditions. The photon flux density was changed successively to 0, 50, 100, 150, 200, 400, 600, 800, 1200, and 1600 μmol m^−2^ s^−1^, and the CO_2_ assimilation rate was recorded after 5 min exposure to each light intensity.

#### Controlled stress condition (growth chamber and water bath)

To compare the photosynthetic capacity of green and red leaves under stress conditions, the Fv/Fm was measured in leaves after dark incubation for 30 min at the required temperature using a chlorophyll fluorescence measuring device (Imaging-PAM, Walz, Effeltrich, Germany). Two types of high temperature stress were undertaken, long-term and short-term. For the long-term stress experiments, plants grown for 1 month in a greenhouse were transferred to a temperature-controlled growth chamber (35°C, 60% relative humidity, 24-hour light period, 400 μmol m^−2^ s^−1^ light intensity, 400 μmol mol^−1^ CO_2_ concentration) for 3 days. One-week-old plants were grown for 1 month in a greenhouse and then transferred to a temperature-controlled growth chamber (35°C, 60% relative humidity, 16-hour light/8-hour dark, 100 μmol m^−2^ s^−1^ light intensity, 400 μmol mol^−1^ CO_2_ concentration) for 2 weeks. The Fv/Fm was measured without and after the plants were subjected to these conditions.

For the short-term stress experiments, water baths were used to quantify the effects of high temperature on the photosynthetic capacity of plants (*58*). The leaves in plants grown for 1 month in a greenhouse were floated in a plastic container filled with water, and the containers were then placed in a heating device (Hot Stirrer, AS ONE, Osaka) or a cooling device (Cool Plate, SCINICS, Tokyo). The Fv/Fm of each leaf was determined without and with 3 and 6 hours of treatment with intense light stress (1000 μmol m^−2^ s^−1^) with a 5700 K LED (ELIXIA, Heriospectra, Göteborg, Sweden) while maintaining each water bath temperature.

### Population genetic analysis

Of the individuals observed for landscape scale around the Tokyo metropolis, a total of 181 individuals from 18 populations were collected, and their genomic DNA was extracted from leaves with the sbeadex Plant DNA Purification Kit on oKtopure, beads-based automated DNA extraction system (LGC Biosearch Technologies, Hoddesdon, UK). To obtain genome-wide SNPs, double-digest restriction site–associated DNA sequencing (ddRAD-seq) was performed (*59*). A ddRAD-seq library was prepared from the genomic DNA digested with the restriction enzymes Pst I and Msp I, as described in (*60*), and sequenced on a DNBSEQ-G400 (MGI Tech, Shenzhen, China) instrument (paired-end 100 bp). Low-quality (<QV15) sequences and adapter sequences were trimmed using Trimmomatic 0.39 (*61*).

The individuals with less than one million reads were removed (*n* = 45). The remaining high-quality reads (*n* = 136) were assembled using stacks v2.4 (*62*) (with commands of “ustacks,” “cstacks,” and “sstacks” parameters of -m = 3, -n = 3, -M = 3, and -N = 5) followed by SNP calling (with “gstacks” of default parameters) and filtering (with “populations” parameters of -R = 0.7, --min-mac = 3, --max-obs-het = 0.6, and --write-random-snp). Pairwise Fst as the genetic distance between populations was calculated by populations command in stacks. The correlation between geographic distance and linearized Fst [Fst/(1-Fst)] as the genetic distance was tested using the vegan package in the R software (*63*). PCA was performed using PLINK ver. 1.9 (*64*). The population genetic structure of individuals was estimated using Admixture ver. 1.3.0 (*65*). Phylogenetic relationships were estimated by the maximum likelihood method based on the GTRGAMMA model with an ascertainment bias correction (*66*) and 1000 bootstrap replicates using RAxML ver. 8.2.12 (*67*). Then, homozygous and variant sites were used in this study. The phylogenetic tree was visualized using ggtree software (*68*).

### Statistical analysis

For all analyses, generalized linear models and generalized linear mixed models were used with software R (*69*, *70*). The details of the statistical models used for each observation and experiment are summarized in table S4. The likelihood ratio test was used to determine the significance of the results using package “car.”

## Supplementary Materials

### This PDF file includes:

Figs. S1 to S9 Legends for tables S1 to S4 References
