# Supplementary material for: From green to red: Urban heat stress drives leaf color evolution
Source: Sci Adv. 2023 Oct 20;9(42):eabq3542. doi: 10.1126/sciadv.abq3542 (PMC10588939; doi:10.1126/sciadv.abq3542)
Supplement: Supplementary file 1 — Figs. S1 to S9 Legends for tables S1 to S4 References [file sciadv.abq3542_sm.pdf]

Science Advances
AAAS

Supplementary Materials for

From green to red: Urban heat stress drives leaf color evolution

Yuya Fukano *et al.*

Corresponding author: Yuya Fukano, yuya.fukano@gmail.com

Sci. Adv. **9**, eabq3542 (2023)
DOI: 10.1126/sciadv.abq3542

This PDF file includes:

Figs. S1 to S9
Legends for tables S1 to S4
References

Other Supplementary Material for this manuscript includes the following:

Tables S1 to S4

Figure S1

Anthocyanin (upper panels) and chlorophyll (lower panels) content of green- and red-leaved *O. corniculate* grown under three growing conditions. (1) non-stress growth chamber condition; grown for 4 weeks at 25°C with 60% humidity, and 400 ppm CO_2 under a 16 h light/8 h dark photoperiod and a light intensity of 150 µmol m^{-2} s^{-1}, (2) non-stress greenhouse condition; plants grown 3 months in greenhouse at ISAS, Tokyo University, with temperature range 15-30°C, relative humidity 30-70 %, and maximum light intensity of 1,500 µmol m^{-2} s^{-1}, (3) stressed brick pavement condition; plants grown for 6 weeks on the experimental brick pavement with maximum ground surface temperatures reaching 50°C, beginning July 26, 2021. Chlorophyll and anthocyanin extraction and quantification were performed according to Nakata & Ohme-Takagi (2014) (*71*).

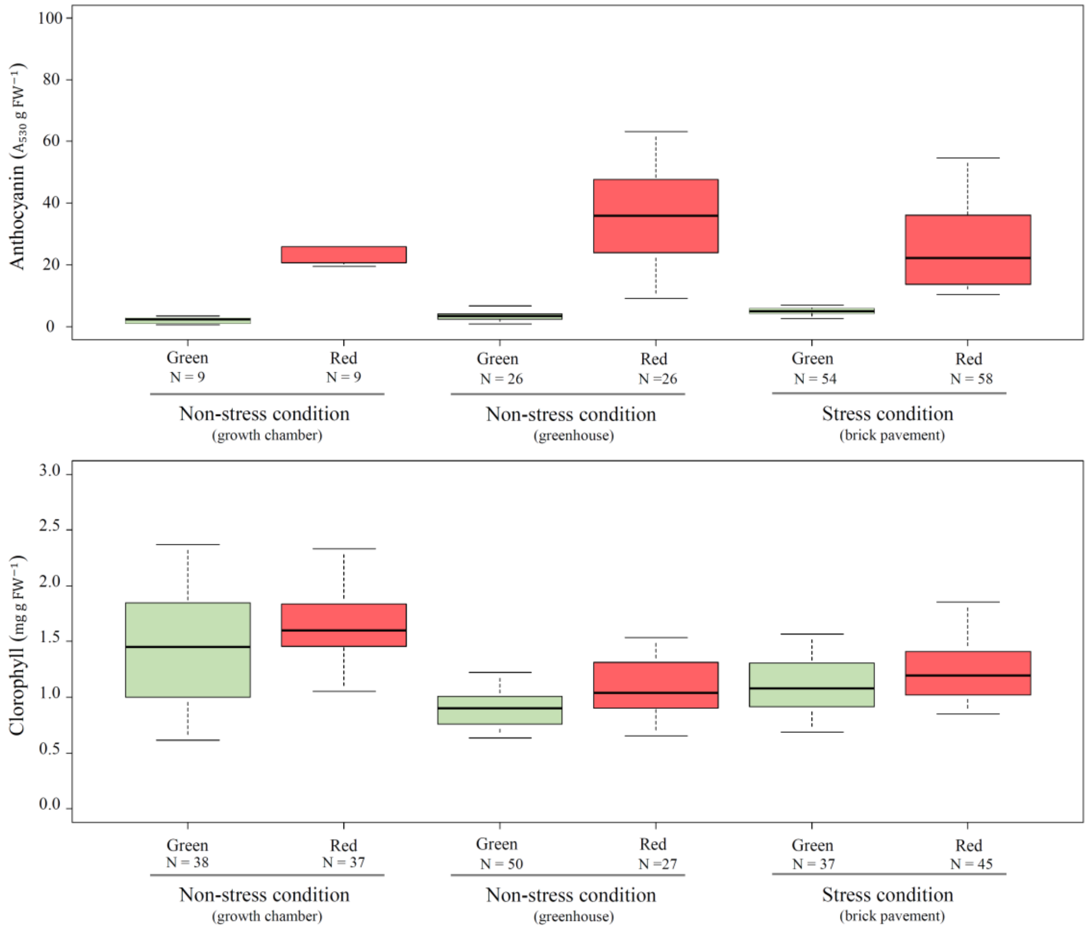

Figure S2

An example of surface temperature comparison between urban and green space in Tokyo. The white square in the photo is survey locations for local-scale belt transect. Data was obtained from Landsat (*72*).

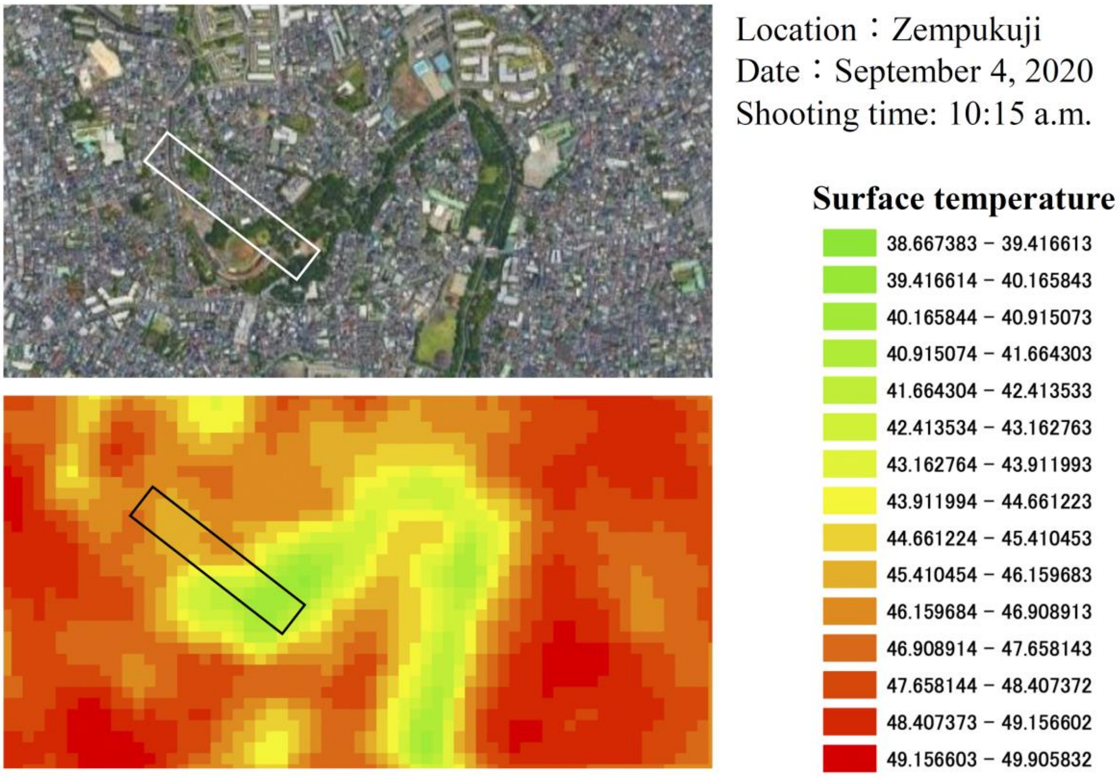

Figure S3

Comparison of total dry weight of green- and red-leaved plants after seven-weeks cultivation under non-stress growth chamber condition. Box plots represent median (horizontal line), 25th and 75th percentile (box), 10th and 90th percentile (whiskers), and each dot represents an individual value.

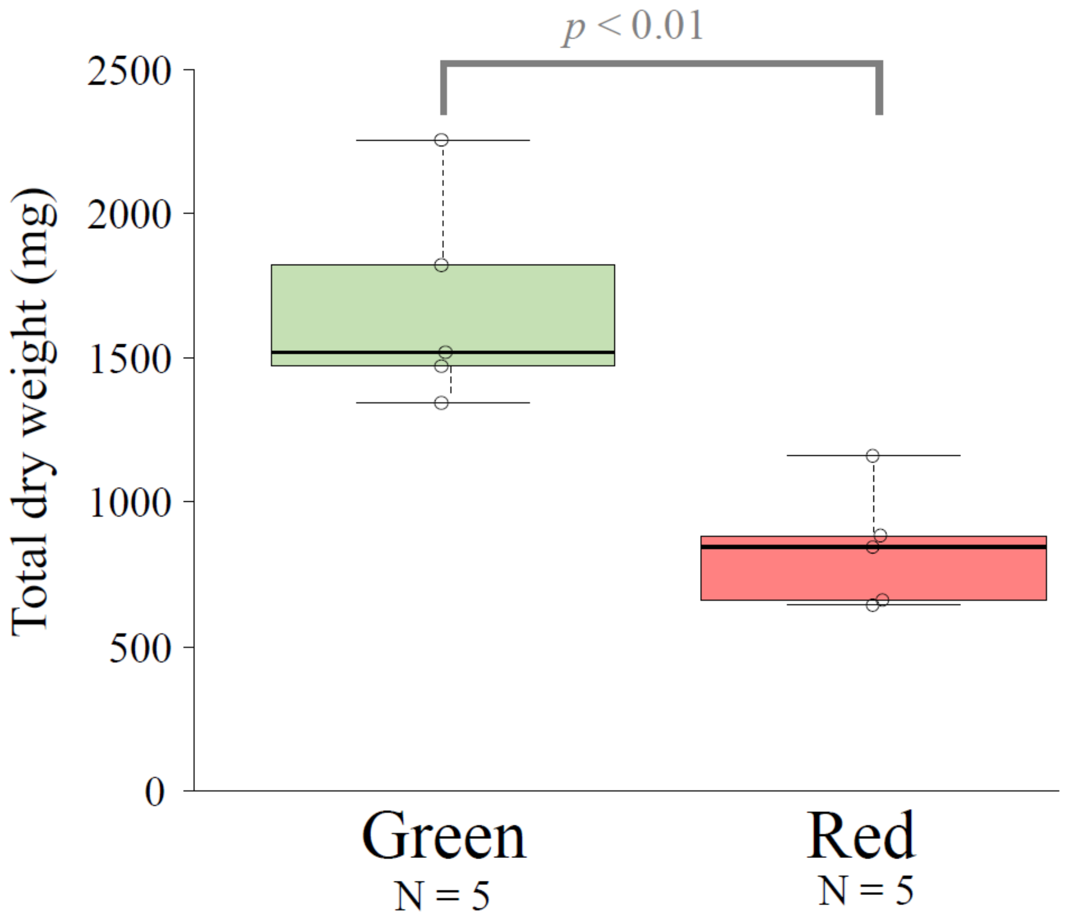

Figure S4

Examples of green- and red-leaved individuals. Pictures shows individuals without (left) and after (right) controlled heat stress experiment.

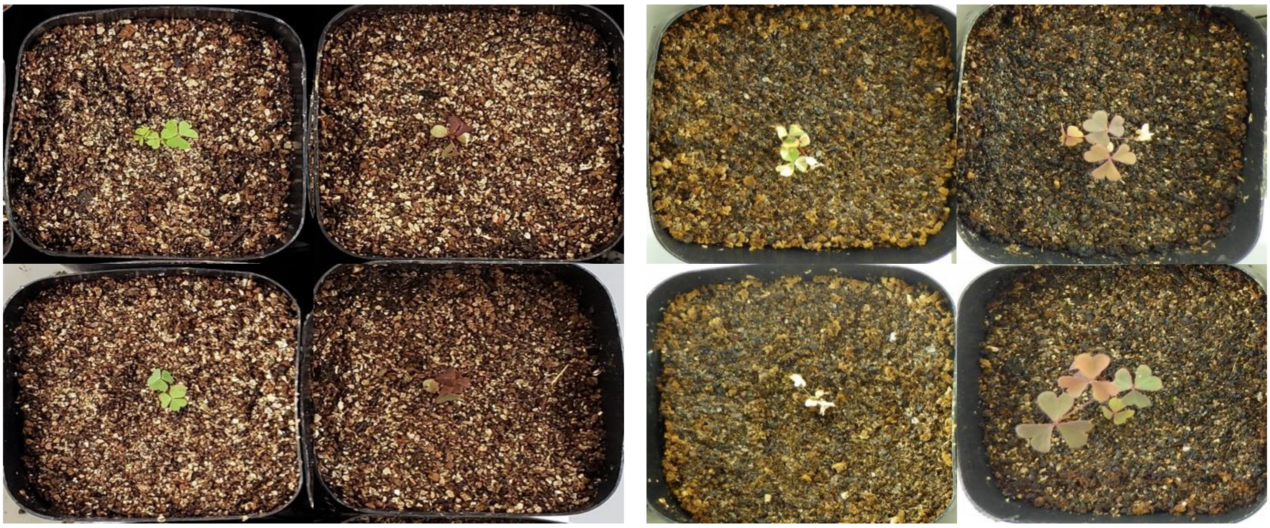

Figure S5

Comparison of maximum photochemical efficiency of photosystem II (Fv/Fm) between green and red leaves without stress treatment. Box plots represent median (horizontal line), 25th and 75th percentile (box), 10th and 90th percentile (whiskers), and each dot represents an individual value.

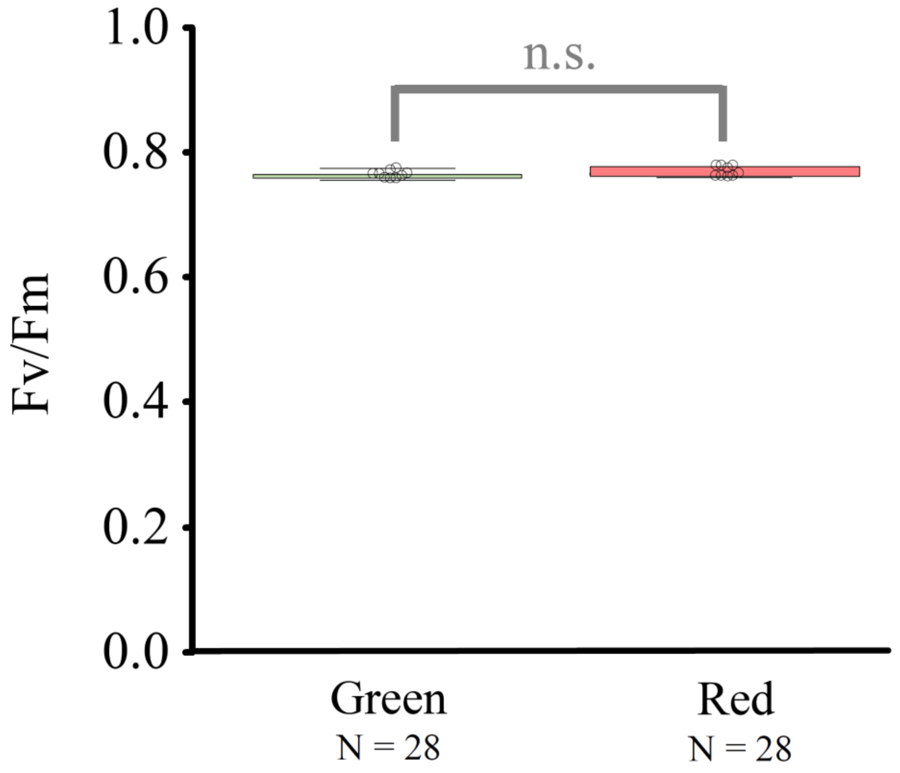

Figure S6

Comparison of maximum photochemical efficiency of photosystem II (Fv/Fm) between green and red leaves after cold treatment in water bath. Box plots represent median (horizontal line), 25th and 75th percentile (box), 10th and 90th percentile (whiskers), and each dot represents an individual value.

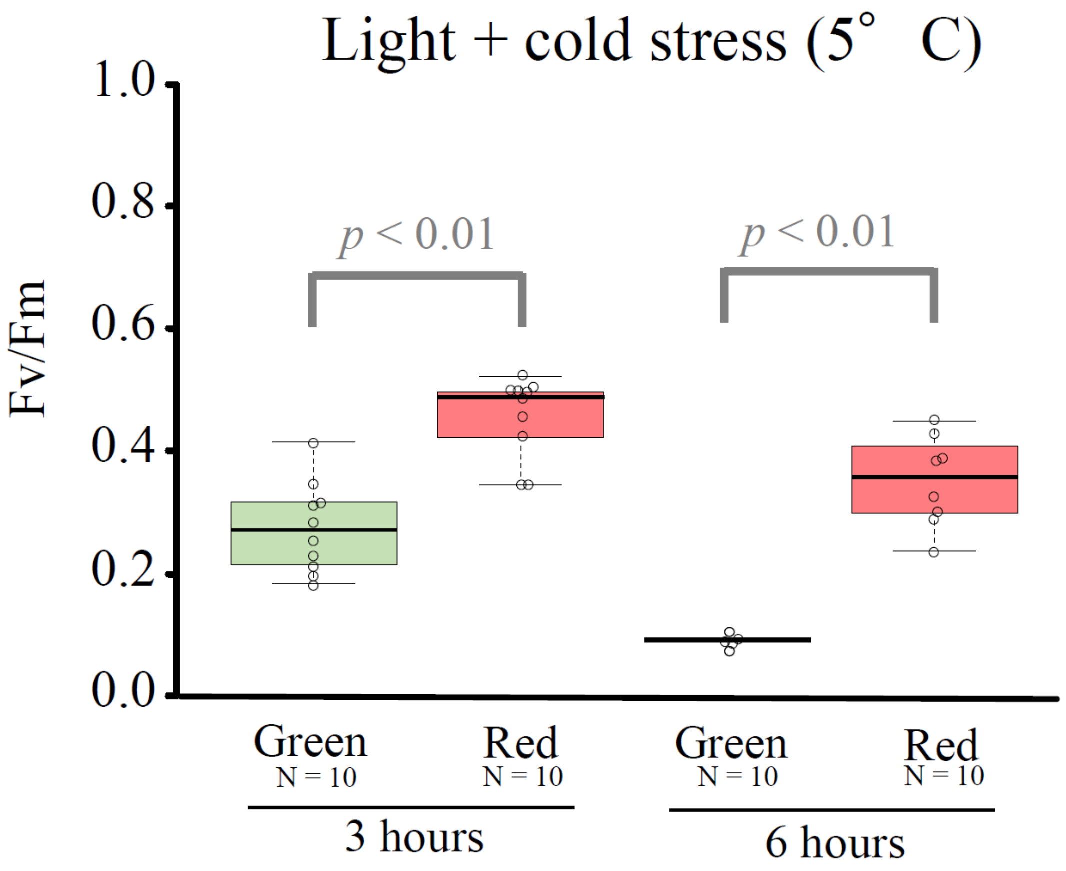

Figure S7

Population genetic structure of *Oxalis corniculata* around the Tokyo metropolis. (A) Principal component analysis. Axis 1 explains 76.67% of the variance, and Axis 2 explains 6.26% of the variance. (B) Relationship between genetic and geographic distance. Genetic distance was calculated by linearized F_{st} ($F_{st}/(1-F_{st})$). (C) Cross-validation error based on ADMIXTURE analysis. The number of clusters (K) is 2 to 18. (D) Clustering assignments at inferred K values of 6 and 7. Each individual is represented by a single vertical line divided into K-colored segments. The bottom color bars indicate population ID, habitat type, leaf color, and clade constructed by phylogenetic analysis. The colors are consistent with Figure 4.

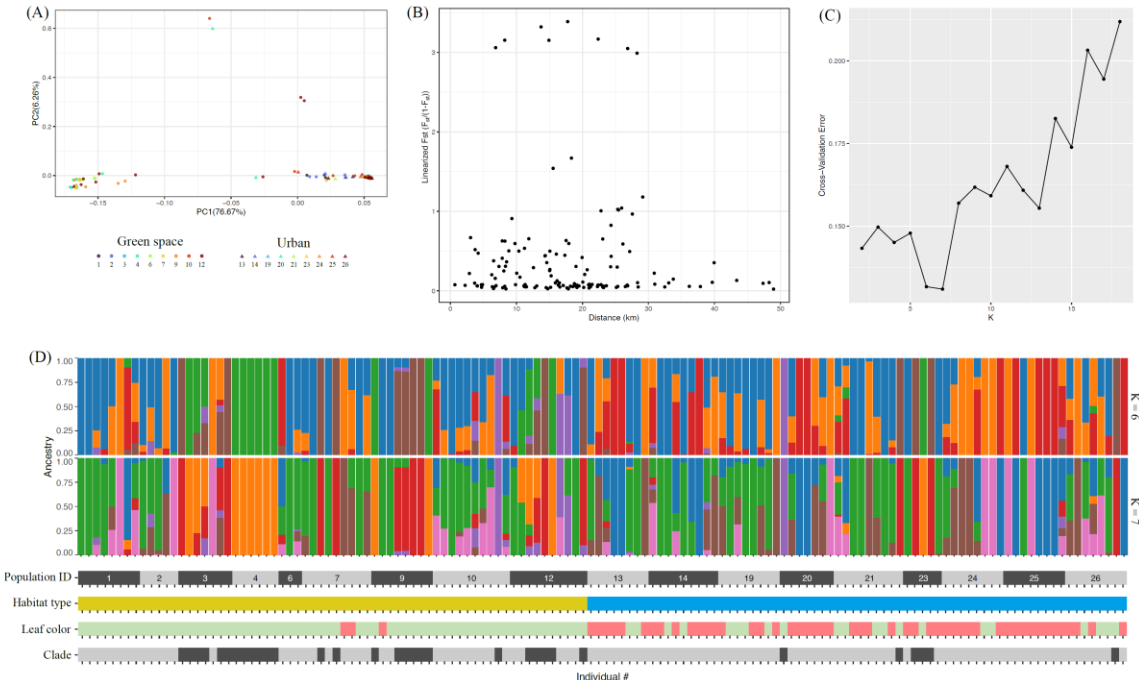

Figure S8

Representative pictures of color scale used for leaf color standards during field observations.

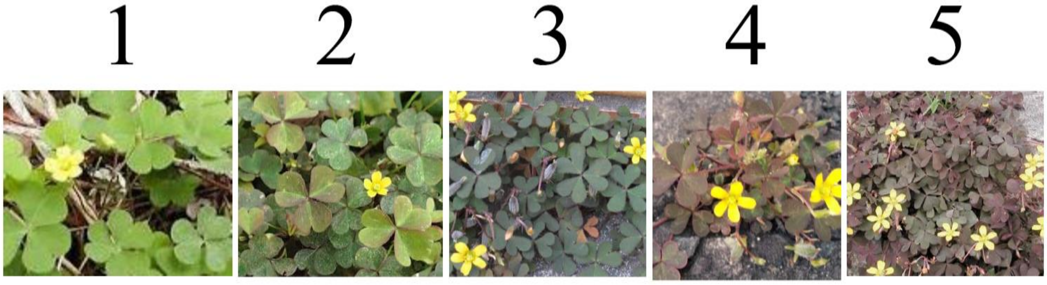

Figure S9

The relationships between five-point visual scale for the leaf color of *Oxalis corniculate* and anthocyanin contents were measured by two different methods. (A) Anthocyanins content extracted from the leaf was calculated from the absorbance at 530 and 637 nm using a spectrophotometer (*71*). (B) Anthocyanin content was measured using an anthocyanin content meter (ACM-200 Plus, Einex Corporation, Tokyo, Japan).

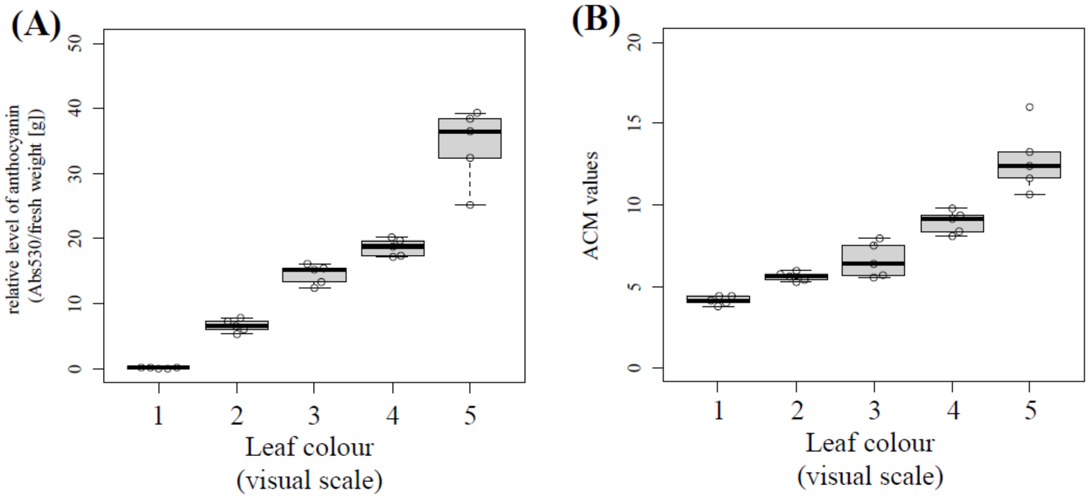

Table S1

The summary of statistical analyses.

Table S2

The survey locations for landscape level observations.

Table S3

The pairwise comparisons of Fst among populations.

Table S4

The summary of the statistical models.

REFERENCES AND NOTES

1. S. H. Faeth, C. Bang, S. Saari, Urban biodiversity: Patterns and mechanisms. *Ann. N. Y. Acad. Sci.* **1223**, 69–81 (2011).

2. M. T. J. Johnson, J. Munshi-South, Evolution of life in urban environments. *Science* **358**, eaam8327 (2017).

3. K. Uchida, H. Fujimoto, A. Ushimaru, Urbanization promotes the loss of seasonal dynamics in the semi-natural grasslands of an East Asian megacity. *Basic Appl Ecol.* **29**, 1–11 (2018).

4. J. S. Santangelo, L. Ruth Rivkin, M. T. J. Johnson, The evolution of city life. *Proc Royal Soc B: Biol Sci*. **285**, 20181529 (2018).

5. L. R. Rivkin, J. S. Santangelo, M. Alberti, M. F. J. Aronson, C. W. de Keyzer, S. E. Diamond, M. J. Fortin, L. J. Frazee, A. J. Gorton, A. P. Hendry, Y. Liu, J. B. Losos, J. S. MacIvor, R. A. Martin, M. J. McDonnell, L. S. Miles, J. Munshi-South, R. W. Ness, A. E. M. Newman, M. R. Stothart, P. Theodorou, K. A. Thompson, B. C. Verrelli, A. Whitehead, K. M. Winchell, M. T. J. Johnson, A roadmap for urban evolutionary ecology. *Evol Appl.* **12**, 384–398 (2019).

6. M. R. Lambert, K. I. Brans, S. Des Roches, C. M. Donihue, S. E. Diamond, Ecology & evolution adaptive evolution in cities: Progress and misconceptions. *Trends Ecol. Evol.*, **1–19**, 239–257 (2020).

7. J. S. Santangelo, R. W. Ness, B. Cohan, C. R. Fitzpatrick, S. G. Innes, S. Koch, L. S. Miles, S. Munim, P. R. Peres-neto, C. Prashad, A. T. Tong, W. E. Aguirre, P. O. Akinwole, M. Alberti, J. Álvarez, J. T. Anderson, Global urban environmental change drives adaptation in white clover *Science* **375**, 1275–1281 (2022).

8. C. J. Schell, K. Dyson, T. L. Fuentes, S. Des Roches, N. C. Harris, D. S. Miller, C. A. Woelfle-Erskine, M. R. Lambert, The ecological and evolutionary consequences of systemic racism in urban environments. *Science*. **369** (2020).

9. M. Alberti, E. P. Palkovacs, S. Des Roches, L. De Meester, K. I. Brans, L. Govaert, N. B. Grimm, N. C. Harris, A. P. Hendry, C. J. Schell, M. Szulkin, J. Munshi-South, M. C. Urban, B. C. Verrelli, The complexity of urban eco-evolutionary dynamics. *Bioscience* **70**, 772–793 (2020).

10. Z. T. Wood, E. P. Palkovacs, B. J. Olsen, M. T. Kinnison, The importance of eco-evolutionary potential in the Anthropocene. *Bioscience* **71**, 805–819 (2021).

11. S. Des Roches, K. I. Brans, M. R. Lambert, L. R. Rivkin, A. M. Savage, C. J. Schell, C. Correa, L. De Meester, S. E. Diamond, N. B. Grimm, N. C. Harris, L. Govaert, A. P. Hendry, M. T. J. Johnson, J. Munshi-South, E. P. Palkovacs, M. Szulkin, M. C. Urban, B. C. Verrelli, M. Alberti, Socio-eco-evolutionary dynamics in cities. *Evol Appl.* **14**, 248–267 (2021).

12. Y. Fukano, Y. Tachiki, M. Kasada, K. Uchida, Evolution of competitive traits changes species diversity in a natural field. *Proc. Royal Soc. B: Biol. Sci.* **289**, 20221376 (2022).

13. J. Verheyen, N. Tüzün, R. Stoks, Using natural laboratories to study evolution to global warming: Contrasting altitudinal, latitudinal, and urbanization gradients. *Curr Opin Insect Sci.* **35**, 10–19 (2019).

14. A. McGaughran, R. Laver, C. Fraser, Evolutionary responses to warming. *Trends Ecol. Evol.* **36**, 591–600 (2021).

15. Z. Liu, C. He, Y. Zhou, J. Wu, How much of the world’s land has been urbanized, really? A hierarchical framework for avoiding confusion. *Landsc. Ecol.* **29**, 763–771 (2014).

16. Q. Yang, X. Huang, J. Yang, Y. Liu, The relationship between land surface temperature and artificial impervious surface fraction in 682 global cities: Spatiotemporal variations and drivers. *Environ. Res. Lett.* **16**, 024032 (2021).

17. W. Kuang, Mapping global impervious surface area and green space within urban environments. *Sci China Earth Sci.* **62**, 1591–1606 (2019).

18. T. R. Oke, “Atmospheric Environment Pergamon Press” (1973).

19. C. Heaviside, H. Macintyre, S. Vardoulakis, The urban heat island: Implications for health in a changing environment. *Curr Environ Health Rep.* **4**, 296–305 (2017).

20. Y. Song, F. Li, X. Wang, C. Xu, J. Zhang, X. Liu, H. Zhang, The effects of urban impervious surfaces on eco-physiological characteristics of Ginkgo biloba: A case study from Beijing, China, *Urban For Urban Green.* **14**, 1102–1109 (2015).

21. S. C. Zipper, J. Schatz, A. Singh, C. J. Kucharik, P. A. Townsend, S. P. Loheide, Urban heat island impacts on plant phenology: Intra-urban variability and response to land cover. *Environ. Res. Lett.* **11**, 054023 (2016).

22. A. L. Hamblin, E. Youngsteadt, M. M. López-Uribe, S. D. Frank, Physiological thermal limits predict differential responses of bees to urban heat-island effects. *Biol. Lett.* **13**, 20170125 (2017).

23. M. S. Fenoglio, A. Calviño, E. González, A. Salvo, M. Videla, "Urbanisation drivers and underlying mechanisms of terrestrial insect diversity loss in cities" in Ecological Entomology (Blackwell Publishing Ltd, 2021), vol. 46, pp. 757–771.

24. S. E. Diamond, L. D. Chick, A. Perez, S. A. Strickler, R. A. Martin, Evolution of thermal tolerance and its fitness consequences: Parallel and non-parallel responses to urban heat islands across three cities in *Proceedings of the Royal Society B: Biological Sciences* (Royal Society Publishing, 2018), vol. 285.

25. S. C. Campbell-Staton, J. P. Velotta, K. M. Winchell, Selection on adaptive and maladaptive gene expression plasticity during thermal adaptation to urban heat islands. *Nat. Commun.* **12**, 6195 (2021).

26. S. C. Campbell-Staton, K. M. Winchell, N. C. Rochette, J. Fredette, I. Maayan, R. M. Schweizer, J. Catchen, Parallel selection on thermal physiology facilitates repeated adaptation of city lizards to urban heat islands. *Nat Ecol Evol.* **4**, 652–658. (2020).

27. R. A. Martin, L. D. Chick, M. L. Garvin, S. E. Diamond, In a nutshell, a reciprocal transplant experiment reveals local adaptation and fitness trade-offs in response to urban evolution in an acorn-dwelling ant. *Evolution (N Y).* **75**, 876–887 (2021).

28. A. Sato, Y. Takahashi, Responses in thermal tolerance and daily activity rhythm to urban stress in Drosophila suzukii. *Ecol. Evol.* **12**, e9616 (2022).

29. T. Merckx, L. Pettersson, M. B. Nielsen, H. J. E. M. Kuussaari, J. Pöyry, J. Tiainen, K. Gotthard, S. M. Kivelä, Urbanization extends flight phenology and leads to local adaptation of seasonal plasticity in Lepidoptera. *Proc. Natl. Acad. Sci.*, **118**, e2106006118 (2021).

30. Y. Chen, X. Wang, B. Jiang, Z. Wen, N. Yang, L. Li, Tree survival and growth are impacted by increased surface temperature on paved land. *Landsc Urban Plan.* **162**, 68–79 (2017).

31. X. M. Wang, X. K. Wang, Y. B. Su, H. X. Zhang, Land pavement depresses photosynthesis in urban trees especially under drought stress. *Sci. Total Environ.* **653**, 120–130 (2019).

32. A. J. Gorton, D. A. Moeller, P. Tiffin, Little plant, big city: A test of adaptation to urban environments in common ragweed (Ambrosia artemisiifolia). *Proc. Royal Soc. B: Biol. Sci.* **285**, 20180968 (2018).

33. P.-O. Cheptou, O. Carrue, S. Rouifed, Rapid evolution of seed dispersal in an urban environment in the weed *Crepis sancta*. *Proc. Natl. Acad. Sci. U.S.A.* **105**, 3796–9 (2008).

34. P. Pyšek, V. Jarošík, P. E. Hulme, J. Pergl, M. Hejda, U. Schaffner, M. Vilà, A global assessment of invasive plant impacts on resident species, communities and ecosystems: The interaction of impact measures, invading species' traits and environment. *Glob. Chang. Biol.* **18**, 1725–1737 (2012).

35. M. I. Malik, S. Mahmood, G. Yasin, N. Bashir, Oxalis corniculata as a successful lawn weed: A study of morphological variation from contrasting habitats. *Pak J Bot.* **44**, 407–411 (2012).

36. JE. Planchon, Oxalis corniculata L. var. atropurpurea. *Flore des serres et des jardins de l'Europe.* **12**, 46–47 (1857).

37. Q. J. Groom, J. Van Der Straeten, I. Hoste, The origin of Oxalis corniculata L. *PeerJ.* **7**, e6384 (2019).

38. J.-H. B. Hatier, K. S. Gould, "*Anthocyanin Function in Vegetative Organs" in Anthocyanins* (Springer, 2008), pp. 1–19.

39. M. Archetti, T. F. Döring, S. B. Hagen, N. M. Hughes, S. R. Leather, D. W. Lee, S. Lev-Yadun, Y. Manetas, H. J. Ougham, P. G. Schaberg, H. Thomas, Unravelling the evolution of autumn colours: An interdisciplinary approach. *Trends Ecol. Evol.* **24**, 166–173 (2009).

40. N. M. Hughes, Winter leaf reddening in "evergreen" species. *New Phytol.* **190**, 573–581 (2011).

41. A. H. Naing, C. K. Kim, Abiotic stress-induced anthocyanins in plants: Their role in tolerance to abiotic stresses. *Physiol. Plant.* **172**, 1711–1723 (2021).

42. N. M. Hughes, S. Lev-Yadun, Review: Why do some plants have leaves with red or purple undersides? *Environ. Exp. Bot.* **205**, 105126 (2023).

43. G. Agati, L. Guidi, M. Landi, M. Tattini, Anthocyanins in photoprotection: Knowing the actors in play to solve this complex ecophysiological issue. *New Phytol.* **232**, 2228–2235 (2021).

44. K. Das, A. Roychoudhury, Reactive oxygen species (ROS) and response of antioxidants as ROS-scavengers during environmental stress in plants. *Front. Environ. Sci.* **2**, 00053 (2014).

45. I. Iosub, F. Kajzar, M. Makowska-Janusik, A. Meghea, A. Tane, I. Rau, "Electronic structure and optical properties of some anthocyanins extracted from grapes" in Optical Materials (Elsevier B.V., 2012), vol. 34, pp. 1644–1650.

46. Y. Fukano, K. Uchida, Y. Tachiki, Urban-rural gradients: How landscape changes drive adaptive evolution of plant competitive traits. Evol Ecol (2023).

47. J. P. Grime, Vegetation classification by reference to strategies. *Nature* **250**, 26–31 (1974).

48. E. Kaibara, Yamato Honzo, Kyotoshorin Nagata chobe (1709), vol. 9; https://rmda.kulib.kyoto-u.ac.jp/item/rb00022856.

49. T. Muranaka, I. Washitani, Alien plant invasions and gravelly floodplain vegetation of the Kinu River. *Ecol. civil Eng.* **4**, 121–132 (2001).

50. S. Takahashi, N. Murata, How do environmental stresses accelerate photoinhibition? *Trends Plant Sci.* **13**, 178–182 (2008).

51. S. O. Neill, K. S. Gould, Anthocyanins in leaves: Light attenuators or antioxidants? *Funct. Plant Biol.* **30**, 865–873 (2003).

52. R. M. Smillie, S. E. Hetherington, Photoabatement by anthocyanin shields photosynthetic systems from light stress. *Photosynthetica.* **36**, 451–463 (1999).

53. K. S. Gould, J. McKelvie, K. R. Markham, Do anthocyanins function as antioxidants in leaves? Imaging of H_2O_2 in red and green leaves after mechanical injury. *Plant Cell Environ.* **25**, 1261–1269 (2002).

54. S. Lev-Yadun, The phenomenon of red and yellow autumn leaves: Hypotheses, agreements and disagreements. *J. Evol. Biol.* **35**, 1245–1282 (2022).

55. W. D. Hamilton, S. P. Brown, Autumn tree colours as a handicap signal. *Proc. Royal Soc. B: Biol. Sci.* **268**, 1489–1493 (2001).

56. M. J. Raupp, P. M. Shrewsbury, D. A. Herms, Ecology of herbivorous arthropods in urban landscapes. *Annu. Rev. Entomol.* **55** (2010), pp. 19–38.

57. C. Wu, A. T. Murray, Estimating impervious surface distribution by spectral mixture analysis. *Remote Sens. Environ.* **84**, 493–505 (2003).

58. L. Shao, Z. Shu, S.-L. Sun, C.-L. Peng, X.-J. Wang, Z.-F. Lin, S. S-l, P. C-l, W. X-j, L. Z-f, Antioxidation of anthocyanins in photosynthesis under high temperature Stress. *J. Integr. Plant Biol.* **49**, 1341–1351 (2007).

59. B. K. Peterson, J. N. Weber, E. H. Kay, H. S. Fisher, H. E. Hoekstra, Double digest RADseq: An inexpensive method for de novo SNP discovery and genotyping in model and non-model species. *PLOS ONE* **7**, e37135 (2012).

60. K. Shirasawa, H. Hirakawa, S. Isobe, Analytical workflow of double-digest restriction site-associated DNA sequencing based on empirical and in silico optimization in tomato. *DNA Res.* **23**, 145–153 (2016).

61. A. M. Bolger, M. Lohse, B. Usadel, Trimmomatic: A flexible trimmer for Illumina sequence data. *Bioinformatics* **30**, 2114–2120 (2014).

62. N. C. Rochette, A. G. Rivera-Colón, J. M. Catchen, Stacks 2: Analytical methods for paired-end sequencing improve RADseq-based population genomics. *Mol. Ecol.* **28**, 4737–4754 (2019).

63. J. Oksanen, F. G. Blanchet, R. Kindt, P. Legendre, P. R. Minchin, R. B. O'hara, M. J. Oksanen, Package 'vegan' (2013).

64. C. C. Chang, C. C. Chow, L. C. A. M. Tellier, S. Vattikuti, S. M. Purcell, J. J. Lee, Second-generation PLINK: Rising to the challenge of larger and richer datasets. *Gigascience.* **4**, 7 (2015).

65. D. H. Alexander, J. Novembre, K. Lange, Fast model-based estimation of ancestry in unrelated individuals. *Genome Res.* **19**, 1655–1664 (2009).

66. P. O. Lewis, A Likelihood Approach to Estimating Phylogeny from Discrete Morphological Character Data (2001), (available at https://academic.oup.com/sysbio/article/50/6/913/1628902), 925.

67. A. Stamatakis, RAxML version 8: A tool for phylogenetic analysis and post-analysis of large phylogenies. *Bioinformatics* **30**, 1312–1313 (2014).

68. G. Yu, D. K. Smith, H. Zhu, Y. Guan, T. T. Y. Lam, ggtree: anrpackage for visualization and annotation of phylogenetic trees with their covariates and other associated data. *Methods Ecol Evol.* **8**, 28–36 (2017).

69. D. Bates, M. Maechler, B. Bolker, S. Walker, R. H. B. Christensen, lme4: Linear mixed-effects models using Eigen and S4 (2014).

70. R Development Core Team, R: A Language and Environment for Statistical Computing. R Foundation for Statistical Computing Vienna Austria (2010), (available at http://mendeley.com/research/r-language-environment-statistical-computing-96).

71. M. Nakata, M. Ohme-Takagi, Quantification of anthocyanin content. *Bio Protoc.* **4**, e1098-e1098. (2014).

72. D. Chen, F. Zhang, M. Zhang, Q. Meng, C. Y. Jim, J. Shi, M. L. Tan, X. Ma, Landscape and vegetation traits of urban green space can predict local surface temperature. *Sci. Total Environ.* **825**, 154006 (2022).
